# Acinar cystic transformation of pancreas as a rare cause of acute pancreatitis and a diagnostic challenge in a young male

**DOI:** 10.1007/s12328-026-02318-z

**Published:** 2026-04-13

**Authors:** Christos Longros, Dietrich Alexander Ruess

**Affiliations:** 1 Department of General and Visceral Surgery, Center for Surgery, Medical Center University of Freiburg, Hugstetter Str. 55, 79106 Freiburg, Germany; 2 German Cancer Consortium (DKTK), Partner Site Freiburg and German Cancer Research Center (DKFZ), Heidelberg, Germany

**Keywords:** Acinar cystic transformation, Pancreatic cyst, Cystic pancreatic lesion, Acute pancreatitis, Pancreaticoduodenectomy

## Abstract

An MRI of a 25-year-old adult male patient after an episode of acute pancreatitis revealed a multiloculated pancreatic head cyst with a mural nodule. Endoscopic biopsy and molecular analysis showed wild-type KRAS, GNAS and TP53. Due to diagnostic uncertainty, a surgical resection was performed and revealed acinar cystic transformation (ACT), a rare benign lesion. ACT should be considered in differential diagnosis of pancreatic cysts, especially in young patients.

## Introduction

Pancreatic cysts frequently present diagnostic and therapeutic challenges. Key clinical difficulty lies in the limited ability to reliably differentiate indolent, benign lesions with serous content—such as serous cystic neoplasms (SCNs) or post-pancreatitis pseudocysts—from mucinous cystic lesions with premalignant potential, including intraductal papillary mucinous neoplasms (IPMNs) and mucinous cystic neoplasms (MCNs). Less common cystic entities like solid pseudopapillary neoplasms (SPNs) or cystic neuroendocrine tumors (cNETs) must also be considered in the differential diagnosis [1].

We here present the rare case of an ACT associated with an episode of acute pancreatitis in a young patient exemplifying these challenges in diagnosis and management of pancreatic cysts.

## Case report

**Fig. 1:**
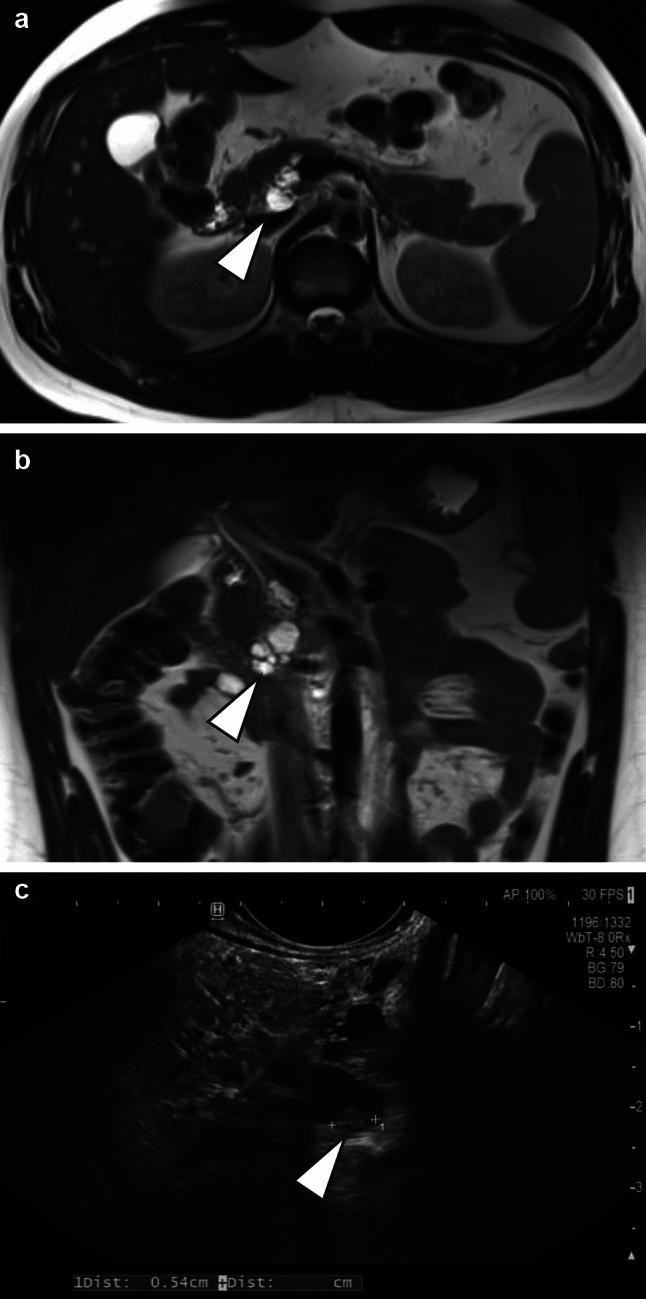
**A** Transverse T2-weighted MRI showing multiloculated cysts of the pancreatic head (arrowhead). **B** Coronal T2-weighted MRI showing multiloculated cysts of the pancreatic head (arrowhead). **C** Endosonography image with arrowhead indicating solid component

A 25-year-old, previously healthy Caucasian male was referred to our surgical outpatient clinic with persistent epigastric and left upper quadrant pain, initiated by an episode of mild acute pancreatitis with elevated serum lipase levels (437 U/l, normal range 7–60 U/l) approximately six months earlier. The constellation had prompted his primary care physician to initiate further investigation. Magnetic resonance imaging had revealed a multiloculated pancreatic cyst located in the head of the pancreas, specifically the uncinate process, overall measuring approximately 3 × 2 × 4 cm, with no involvement or obstruction of the main pancreatic duct or the common bile duct (Fig. 1 A + B, arrowheads). No further CT imaging was performed. The patient reported a positive family history of pancreatic cancer, having affected his mother’s uncle. At the time of presentation, serum lipase remained mildly elevated at 113 U/l. Tumor markers were within normal limits and unremarkable: CA 19–9: 16.7 U/mL (normal < 27 U/mL), CEA: 2.0 ng/mL (normal < 4.7 ng/mL), CA 125: 5.4 U/mL (normal < 35 U/mL), and CA 15–3: 18.6 U/mL (normal < 25 U/mL). There was no indication of endocrine dysfunction (HbA1c: 5.3%, normal: 4.8–5.7%).

We performed endoscopic ultrasound (EUS) which confirmed multiple confluent cysts in the pancreatic head, without main duct dilation. A mural nodule measuring 5.4 mm in width was detected (Fig. 1 C, arrowhead); contrast enhancement was not performed. During the EUS-guided puncture, no fluid could be aspirated despite needle repositioning, although a small amount of tissue was obtained. The same 19G FNA-needle was used for both aspiration and biopsy (Moray™ Micro Biopsy Forceps, STERIS Endoscopy) attempts. The EUS-guided biopsy revealed epithelial strips, adjacent to unremarkable exocrine pancreatic tissue, which were not unequivocally diagnostic. DNA panel exon sequencing demonstrated wild-type genotypes for *GNAS*, *KRAS* and *TP53*.

According to the Kyoto international consensus guidelines for the management of IPMN [2], the presence of multiple worrisome features including a history of acute pancreatitis, overall cyst diameter ≥ 30 mm, and a mural nodule indicated increased risk of high-grade dysplasia or IPMN-associated invasive carcinoma, warranting a recommendation of surgical resection if clinically appropriate.

We therefore engaged in an open and thorough discussion with the patient, carefully weighing the options of surgical resection via pancreatoduodenectomy versus close surveillance with MRI. Together, we considered the trade-off between diagnostic certainty and potential cancer prophylaxis on one hand, and the risks associated with major surgery on the other, including morbidity, mortality, and the long-term consequences of pancreatic resection such as risk for exocrine and endocrine insufficiency.

After informed consent, the patient elected to proceed with surgical management. A minimally invasive, laparoscopic pylorus-preserving pancreatoduodenectomy with Cattell-Warren pancreaticojejunostomy was performed.

Intraoperatively an accessory right hepatic artery supplying Couinaud liver segments 6/7 was accidentally injured. An end-to-end arterial reconstruction was performed via a hybrid laparotomy approach. Due to a high-risk pancreaticojejunostomy—characterized by soft pancreatic tissue, a narrow duct diameter (< 2 mm), and a body mass index of 28 kg/m^2^—an externalized pancreatic duct stent and surgical drains were placed. The postoperative course was complicated by Grade B postoperative pancreatic fistula (POPF-B), and Grade B postpancreatectomy hemorrhage (PPH-B), according to the International Study Group for Pancreatic Surgery (ISGPS) [3, 4], requiring angiographic coiling of the gastroduodenal artery stump. Follow-up over the subsequent year has been unremarkable, with no episodes of pancreatitis, pain or endocrine insufficiency.

**Fig. 2:**
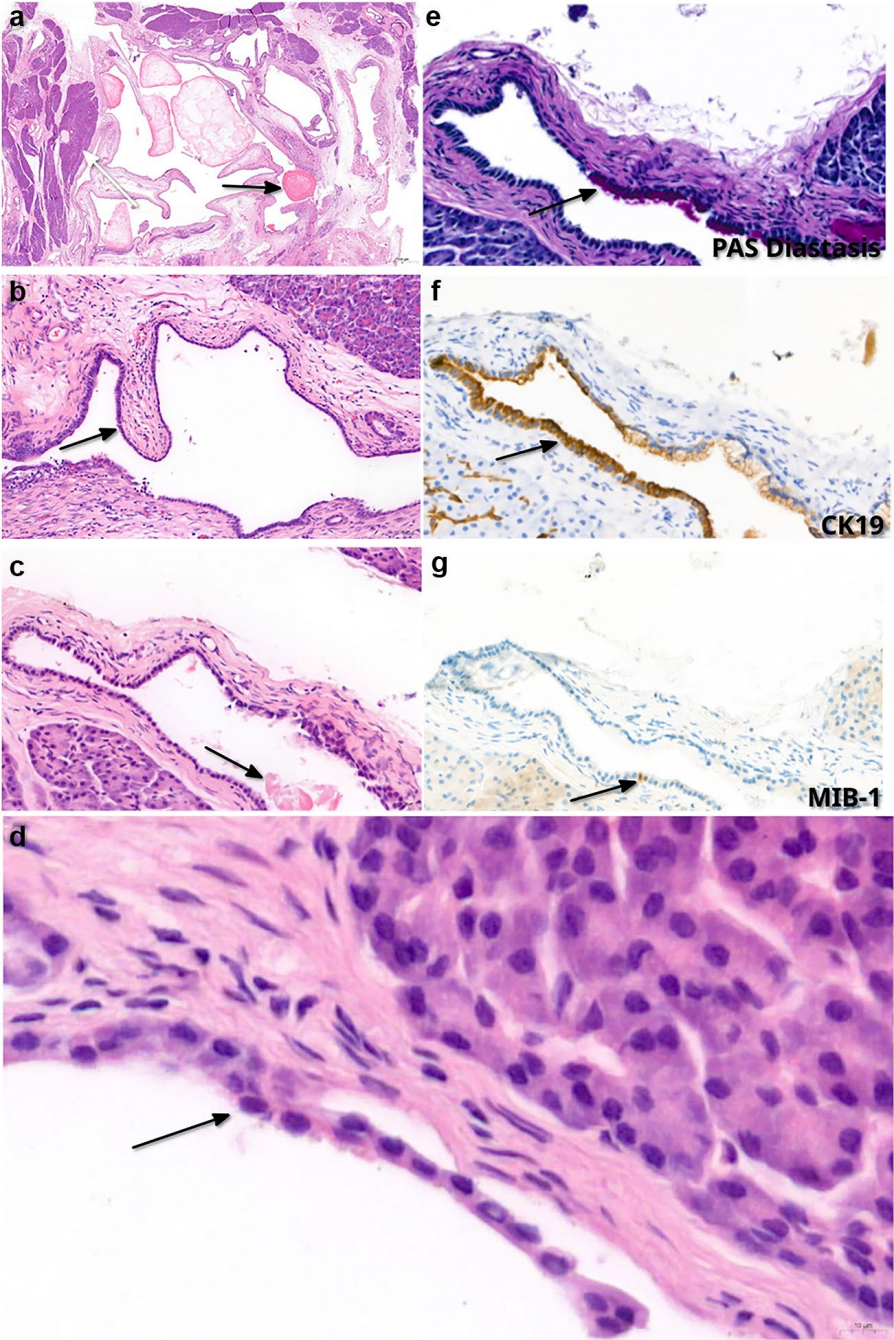
**A** H&E staining showing multiple cystic structures partially surrounded by acinar tissue (white arrow) and areas of fibrosis; cyst lumina contain corpora amylacea (black arrow) (original magnification × 20). **B** H&E staining showing incomplete septation of cyst walls forming club-like pseudopapillary structures (arrow) (original magnification × 100). **C** H&E staining showing cyst lumina containing eosinophilic enzymatic secretions (arrow) (original magnification × 200). **D** H&E staining at high magnification showing cyst wall lined by cuboidal acinar epithelium with characteristic basophilic nuclei and pale cytoplasm; no cytological atypia or mitotic figures are observed (arrow) (original magnification × 400).**E** PAS-diastase staining showing positive diastase-resistant cytoplasmic granules, indicating acinar differentiation (arrow) (original magnification × 200). **F** CK19 immunohistochemistry showing strong expression, indicating ductal differentiation (arrow) (original magnification × 200). **G** MIB-1 immunohistochemistry showing a low proliferation index with < 2% positive fraction (arrow) (original magnification × 200)

Intraoperative cryosectioning confirmed complete resection. The pancreatic parenchyma showed multiple cysts without communication with the pancreatic duct, some containing globular white concretions up to 0.9 cm in diameter. However, the mural nodule observed during preoperative EUS was not identified or further characterized in the pathological examination. Histological sections revealed considerable fibrosis and focal calcifications, which may represent the pathological correlate of the imaging finding. However, a definitive correlation could not be established. Hematoxylin and eosin (H&E) staining confirmed multiple cystic structures, partially surrounded by acinar tissue and areas of fibrosis (Fig. 2 a + b). Cyst lumina contained corpora amylacea and eosinophilic enzymatic secretions (Fig. 2 a + c). Cyst walls exhibited incomplete septation, forming club-like pseudopapillary structures (Fig. 2 b). The cystic epithelial lining was composed of a single layer of cuboidal to columnar cells with pale cytoplasm (Fig. 2 d), some of which exhibited a granular appearance. There was no evidence of mucin production, cytological atypia, mitotic figures, or invasive growth. Histologically, the lesion showed mixed acinar (PAS-positive, diastase-resistant cytoplasmic granules (Fig. 2 e)), and ductal (strong expression of cytokeratin 19 (CK19) (Fig. 2 f) differentiation). Proliferation index was low, with an MIB-1 positive fraction of less than 2% (Fig. 2 g).

Immunohistochemistry was also performed for trypsin, chymotrypsin, CK7, GLUT1 and inhibin. The acinar cells of the dilated acini expressed trypsin and chymotrypsin, CK7 stained the dilated ducts, as CK19. No specific expression of GLUT1 or inhibin was present in the epithelium.

The differential diagnosis of SCN was considered but deemed unlikely due to the absence of glycogen-rich epithelium. Mucinous epithelium characteristic of IPMN was not observed. MCN was excluded based on the absence of ovarian-type stroma. DNA panel exon sequencing once again revealed no mutations in *KRAS*, *GNAS* or *TP53*.

Overall, histopathological and molecular findings were consistent with the diagnosis of acinar cystic transformation (ACT) of the pancreas.

## Discussion

ACT of the pancreas is a rare benign entity, initially termed “acinar cystadenoma”, reflecting the original perception of the lesion as a benign counterpart to acinar adenocarcinoma [5]. Later, cystadenoma with its neoplastic connotation was suggested to be replaced with the term "acinar cystic transformation", to better reflect its benign nature [6].

The majority of ACT lesions present as unifocal, multiloculated cystic structures, with localization occurring equally in the pancreatic head and body-tail regions. There is an approximate 2:1 female predominance, with a median age at diagnosis in the early 40 s. Histologically, the cysts are typically lined by acinar epithelium of either cuboidal or columnar shape, which is interspersed with ductal-like epithelium in about 50% of cases. The epithelium is typically lined by 1–2 cell layers showing a pale-granular cytoplasm that is immunohistochemically positive for trypsin, chymotrypsin and CK7. Cysts also contain amylacea-like acidophilic concretions that represent enzymatic secretions due to the connection to active acini [7, 8]. Features suggestive of malignancy such as nuclear atypia, mitotic figures, necrosis, infiltrative growth, and associated invasive carcinoma are consistently absent. The proliferative index is low, generally around 1–2% [6, 8].

ACT is thought to originate from acinar microcysts, developmental malformations, obstructive-inflammatory processes, or genetic predisposition. A neoplastic origin remains controversial due to the occasional detection of mutations such as KRAS or SMO [6]; however, all reported ACT cases have been benign with no evidence of malignant transformation. Preoperative diagnosis is challenging, with abdominal pain being the most common clinical presentation in approximately 42% of patients, followed by weight loss and a palpable mass. According to a systematic review by Mattiolo et al. [9], approximately 25% of cases are misclassified as unspecified cystic pancreatic lesions.

Since the systematic review by Mattiolo et al. [9], several additional cases of ACT have been reported (Table 1). Among these, the majority presented with nonspecific symptoms such as abdominal pain or were discovered incidentally [10–16]. Preoperative misdiagnosis remained a common challenge, with ACT most frequently confused with IPMN [10–12], followed by mucinous cystic neoplasm [14] and lymphoepithelial cyst [15]. Most lesions were unifocal and multilocular, located predominantly in the pancreatic head [11, 13, 16]. Immunohistochemical confirmation of acinar differentiation with trypsin and/or chymotrypsin positivity was consistent across all cases. Molecular analysis was performed in only a minority of cases; where reported, KRAS and GNAS were wild-type [13, 15], supporting the non-neoplastic nature of ACT. Low-grade PanIN was identified in one case [13], consistent with the 4% incidence reported by Mattiolo et al. [9]. All patients had a benign postoperative course with no evidence of disease at follow-up, further supporting a conservative approach once ACT is histologically confirmed.

**Table 1:**
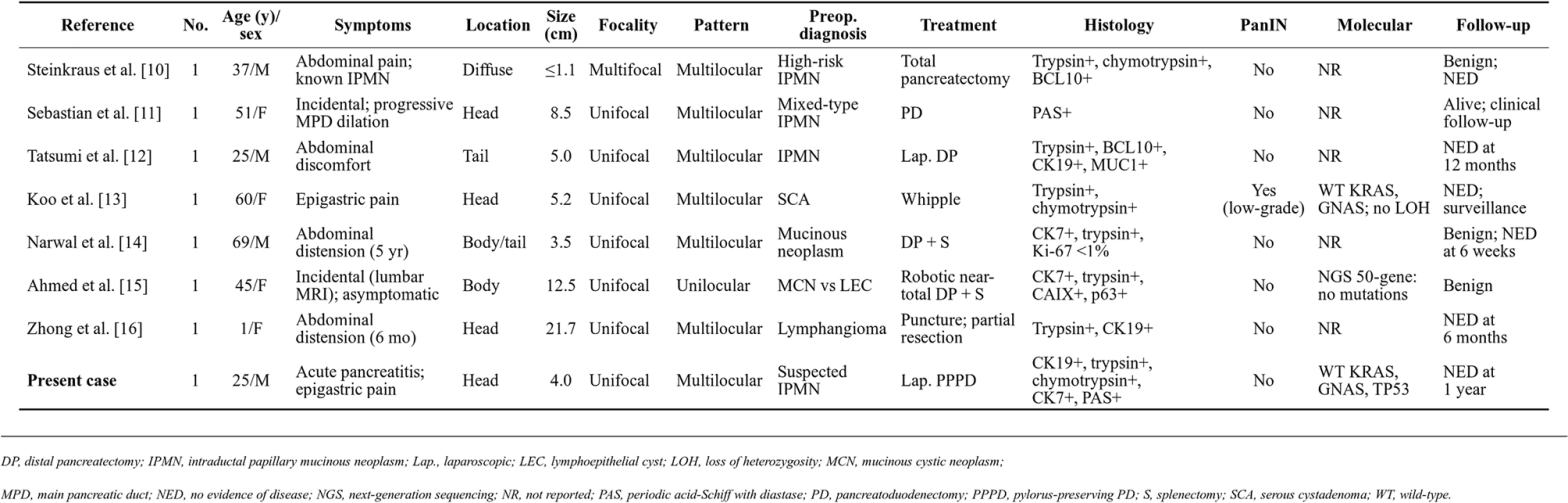
Recently reported cases of acinar cystic transformation of the pancreas (2023–2025), depicting preoperative diagnosis, clinicopathological, immunohistochemical, and molecular characteristics

Pancreatic cysts other than IPMNs that can preoperatively mimic ACTs mainly include serous cystadenomas (SCA) and mucinous cystic neoplasms (MCN) [9].

SCAs are found more frequently in women, during their 5th to 7th decade of life. Most lesions originate in the pancreatic body and tail, with approximately 40% arising in the head. Symptoms like non-specific abdominal pain are uncommon (about 27%). Morphological imaging features include a fibrous central scar with calcification giving a “sunburst” appearance as well signs of hypervascularity [17].

Mucinous cystic neoplasms show a stronger female sex predilection with a female to male ratio of 20:1. The vast majority (93%) of these cysts arise in pancreas body and tail as a solitary multiloculated lesion that is often accompanied by calcification without communication with the pancreatic duct. 62.2% of symptomatic patients have abdominal pain [18].

ACT is not currently mentioned in existing guidelines [2] or in high-impact reviews on pancreatic cysts [1], which adds an extra layer of complexity for clinicians, given the variability and interpretative differences among the three major guidelines available for pancreatic cyst management [19].

To improve non-invasive diagnostic accuracy, imaging analysis algorithms show promise: Recently, four imaging criteria with strong diagnostic value for differentiating ACT from branch-duct IPMN have been proposed: the presence of five or more cysts, clustered peripheral small cysts, cyst calcifications, and absence of communication with the main pancreatic duct [20]. However, these criteria remain insufficient to establish a definitive diagnosis in a subset of patients [21]. Advances in artificial intelligence and radiomics hold potential to enhance diagnostic precision in the near future [22].

In conclusion, increased awareness of ACT as separate pathological entity, supported by inclusion into guidelines as differential diagnosis of cystic lesions of the pancreas may reduce the rate of misdiagnosis and overtreatment. Yet, although imaging features may aid preoperative decision-making, a definitive diagnosis still requires histopathological examination and can usually only be established after surgical resection.
